# *Cis*-regulatory variation expands the colour palette of the Brassicaceae

**DOI:** 10.1093/jxb/erac366

**Published:** 2022-11-02

**Authors:** Róisín Fattorini, Diarmuid S Ó’Maoiléidigh

**Affiliations:** Institute of Systems, Molecular, and Integrative Biology, University of Liverpool, UK; Institute of Systems, Molecular, and Integrative Biology, University of Liverpool, UK

**Keywords:** Anthocyanins, carotenoids, *Brassica napus*, *cis*-regulatory variation, multi-omics, petal colour, R2R3-MYBs

## Abstract

This article comments on:

**Ye S, Hua S, Ma T, Ma X, Chen Y, Wu L, Zhao L, Yi B, Ma C, Tu J, Shen J, Fu T, Wen J.** 2022. Genetic and multi-omics analyses reveal *BnaA07.PAP2*^*In-184-317*^ as the key gene conferring anthocyanin-based color in *Brassica napus* flowers. Journal of Experimental Botany **73,**6630–6645.

**Rapeseed (*Brassica napus*) has recently become a popular ornamental plant in China, moonlighting from its more prominent role as a source of dietary oil. Ye *et al.* (2022) have identified changes in the expression pattern of a gene that encodes an R2R3-MYB transcription factor as causal for petal colour variation in *B. napus*. A multi-omics approach outlines how increased expression of this transcription factor influences anthocyanin production to modify pigmentation independently or in concert with variation in carotenoid pigment biosynthesis.**

Autumnal leaves decorate the landscape with a broad range of vibrant colours, which is determined by the mixture of pigments present in leaves. While leaf pigmentation may protect against abiotic factors or provide defence against biotic stressors (Archetti *et al.*, 2009), an additional prominent role for floral colouration is pollinator attraction. Animal pollination is important for reproductive success in many flowering plant species, and pigmentation, among other factors, can significantly influence flower visitation (Fattorini and Glover, 2020). Furthermore, novel pigmentation and patterns are in high demand in the ornamental plant marketplace (Zhao and Tao, 2015). Petal colour is a quantifiable phenotype that has been used as a vehicle for exploring the mechanisms of genetic, developmental, and evolutionary change. The genes encoding pigment pathway enzymes are well described, and genetic regulators have been identified in many systems (Grotewold, 2006; Tanaka *et al.*, 2008; Davies *et al.*, 2012).

Pigment composition and concentration are primary factors influencing petal appearance. In concert with other factors, variation in the activity of the enzymes responsible for the anabolism or catabolism of these pigments and their intermediates leads to a diversity in petal colours (Tanaka *et al.*, 2008). Elevating carotenoid concentrations normally leads to yellow and orange hues in petals, whereas accumulation of certain flavonoids, such as anthocyanins, can colour petals black, pink, red, purple, or blue. In the absence of these and other pigments, petals tend to be white (Tanaka *et al.*, 2008).

The MBW complex, which contains R2R3-*M*YB and *b*asic helix–loop–helix (bHLH) transcription factors in addition to a *W*D40 repeat domain protein, is a well-characterized regulator of anthocyanin biosynthesis (Grotewold, 2006; Davies *et al.*, 2012). The R2R3-MYB family is one of the largest transcription factor families in flowering plants, and members of this family control development and cell differentiation, responses to biotic and abiotic stress, and specialized metabolism (Wu *et al.*, 2022). The latter includes flavonoid regulation and, contributing to this body of work, Ye *et al.* (2022) investigated the molecular mechanisms within *B. napus* petals underlying anthocyanin pigmentation. The authors also synthesize an understanding of how variation in both anthocyanin and carotenoid biosynthesis leads to diverse petal colours in *B. napus*.

## Variation in the activity of two genes produces four petal colours

The mechanisms determining anthocyanin-based flower colouration in *B. napus* were investigated using apricot-, pink-, white-, and yellow-flowered plants. The progeny of a cross between apricot- and white-flowered plants were self-fertilized and this F_2_ population segregated for each of the four petal colour phenotypes. The segregation ratio indicated that two dominant genes were causal for this colour variation. Pink-flowered plants harboured both dominant alleles, whereas yellow flowers harboured the corresponding recessive alleles. A metabolomics approach confirmed that anthocyanin and carotenoid derivatives were major contributors to divergence in petal colouration (Box 1). A previously known mutation was causal for the divergence in carotenoid levels (Zhang *et al.*, 2015) (Box 1); however, the source of variation in anthocyanin production, termed the *APRICOT FLOWERS* (*APF*) locus, was unclear.

**Box 1. Differential gene expression underlying variation in petal pigmentation in *B. napus***

The MYB–bHLH–WDR (MBW) complex promotes the expression of anthocyanin biosynthesis genes, such as those encoding dihydroflavonol 4-reductase (DFR), anthocyanidin synthase (ANS), and flavonoid 3-*O*-glycosyl-transferase (UFGT). These enzymes are involved in the synthesis of anthocyanins, including cyanidins, among which cyanidin 3-*O*-glucoside was the most differentially abundant compound between white/yellow and pink/apricot petals. Despite containing petunidins/delphinidins, yellow and white petals lacked a purple hue associated with these pigments. The authors speculate that this may be due to the influence of co-pigments on overall petal colouration. Notably, only trace levels of *BnaA07.PAP2* were detected in yellow and white petals although they contain abundant petunidin/delphinidin. This suggests that synthesis of these anthocyanins is promoted through other means, perhaps by an MBW complex containing different MBW homologues. Based on evidence from other species, it is reasonable to suggest that BnaPAP2 acts in the MBW complex; however, this has not been demonstrated. Therefore, it is also possible that PAP2 acts independently of this complex.

Petals of yellow- and apricot-flowered plants had significantly enhanced lutein concentrations, while colourless carotenes were more abundant in white and pink petals. In yellow-flowered plants, an insertion in the coding region of *BnaC3.CCD4* (*CAROTENOID CLEAVAGE DIOXYGENASE 4*) disrupts *CCD4* transcription, enabling α-carotenes to be converted into yellow xanthophylls in the petal. In white-flowered plants, *BnaC3.CCD4* lacks this insertion and is expressed in petals. BnaCCD4 cleaves α-carotenes into colourless volatiles, thus preventing the formation of yellow petal colouration (Zhang *et al.*, 2015). Liu *et al.* (2020) identified an orange-flowered *B. napus* phenotype that resulted from a shift in carotenoid composition, relative to the yellow petals. In the absence of *ZEAXANTHIN EPOXIDASE* (*ZEP*) homologues, lutein accumulates and violaxanthin content in petals is reduced, producing an orange petal phenotype. Evidently, altered expression of very few genes that encode biosynthesis enzymes or pathway regulators is responsible for the striking colour variation between *B. napus* flowers.

WDR, WD40 repeat domain protein; DHQ, dihydroquercetin; DHM, dihydromyricetin; LCC, leucocyanidin; LCD, leucodelphinidin.

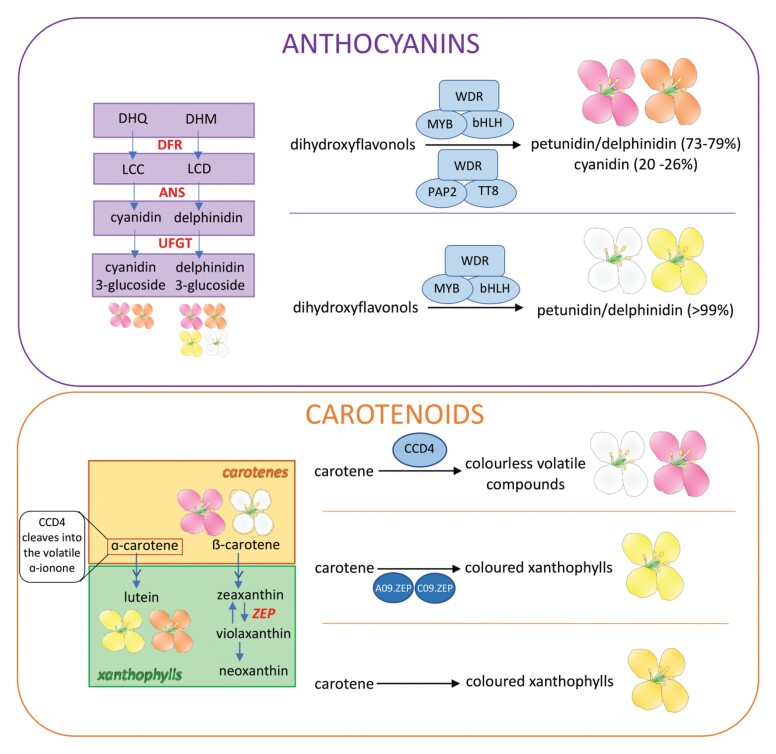

## Petal colour variation explained through a multi-omics approach

A comparative approach incorporating all floral phenotypes enabled identification of genetic commonalities that correlated with shared petal pigmentation phenotypes. Bulked-segregant sequencing-based mapping defined the *APF* locus as occurring in a 365 kb genomic region, while pairwise transcriptome comparisons identified differentially expressed genes between anthocyanic and non-anthocyanic petal phenotypes. *BnaA07.PAP2* was up-regulated in petals from pink- and apricot-flowered plants and is located within the identified genomic interval. This gene encodes an R2R3-MYB transcription factor that is a positive regulator of anthocyanin biosynthesis in *B. napus* leaves (Chen *et al.*, 2020). *BnaA07.PAP2* was highly expressed in petals and buds of apricot-flowered plants but was expressed at very low levels in yellow-flowered plants. In pink- and apricot-flowered plants, *BnaA07.PAP2* promoter regions contained two insertions that were not present in yellow- or white-flowered individuals (Box 2). Integration of the promoter and genomic sequence of *BnaA07.PAP2* derived from apricot-flowered *B. napus* was sufficient to convert the petals of yellow-flowered plants to apricot. Additionally, and in contrast to the *BnaA07.PAP2* promoter from yellow-flowered plants, the *BnaA07.PAP2* promoter from apricot-flowered plants was able to drive the expression of a reporter gene in leaves and flowers. These functional assays confirmed that the colour change from yellow- to apricot-flowered *B. napus* plants occurred due to activation of *BnaA07.PAP2* that resulted from insertions in its promoter region.

## *Cis-*regulatory variation drives petal colour change

Changes in the transcriptional regulation of genes encoding R2R3-MYB transcription factors that influence anthocyanin production have been noted in several other members of the Brassicaceae (Box 2) and in other species. A *cis-*regulatory change in a *Phlox drummondii* R2R3-MYB-encoding gene contributes to petal colour variation through differential anthocyanin production (Hopkins and Rausher, 2011). Pigmentation patterns are also influenced by regulatory variation in genes encoding R2R3-MYB transcription factors. In *Clarkia gracilis*, for example, *cis*-regulatory differences in the promoter region of *CgMyb1* shift the spatial positioning of petal anthocyanin accumulation between subspecies (Martins *et al.*, 2017). Similarly, in *Erythranthe* (formerly *Mimulus* spp.), *cis-*regulatory variation upstream of the *LIGHT AREAS* (*LAR*) is probably causal for modifying its expression pattern in petals between two sister species. LAR promotes the expression of *FLAVANOL SYNTHASE*, leading to the production of colourless flavanols instead of anthocyanins (Yuan *et al.*, 2016). Expression changes in genes encoding R2R3-MYB transcription factors may be widespread because they result in few deleterious pleiotropic effects when ectopically expressed and are functionally specific (Sobel and Streisfeld, 2013).

**Box 2. *Cis-*regulatory variation modifies MYB gene expression in the Brassicaceae**

Several studies have identified variation in the promoter sequences (black lines) of genes that encode R2R3-MYB transcription factors (open boxes) as causal for changes in anthocyanin production. Transposable elements (TEs), such as from the CACTA and Harbinger families of transposons, have been found to enhance transcriptional output (indicated by blue chevrons) (Chiu *et al.*, 2010; Yan *et al.*, 2019). Interestingly, although purple cauliflower lack a TATA box (Δ6 bp, red triangle) relative to white cauliflower as well as another base pair (red triangle), the insertion of the Harbinger TE appears to be sufficient to significantly enhance expression of *BoMYB2* (Chiu *et al.*, 2010). Similarly, although the purple kale and kohlrabi promoters contain several other differences relative to the white cabbage promoter, the CACTA TE appears to be largely responsible for the increased expression in these cultivars (Yan *et al.*, 2019). Very subtle changes in the promoter sequences, whose effects are difficult to predict, can also have a dramatic influence on transcription. Six nucleotide substitutions (green triangles) and a single nucleotide insertion (orange triangle) increased the expression of *BoMYB2* considerably in purple cabbage relative to green cabbage (Yan *et al.*, 2019). Likewise, three nucleotide insertions boost the transcriptional output of *BnaPAP2.A7* in purple rapeseed relative to green rapeseed (Chen *et al.*, 2020). Ye *et al.* (2022) describe insertions that contain a number of CAAT enhancer elements in plants bearing apricot or pink petals of *B. napus*. These examples illustrate the vast different modes of promoter evolution that can influence gene expression outputs to modify pigmentation.

Sequence and expression comparisons are relative to the first cultivar in each box.

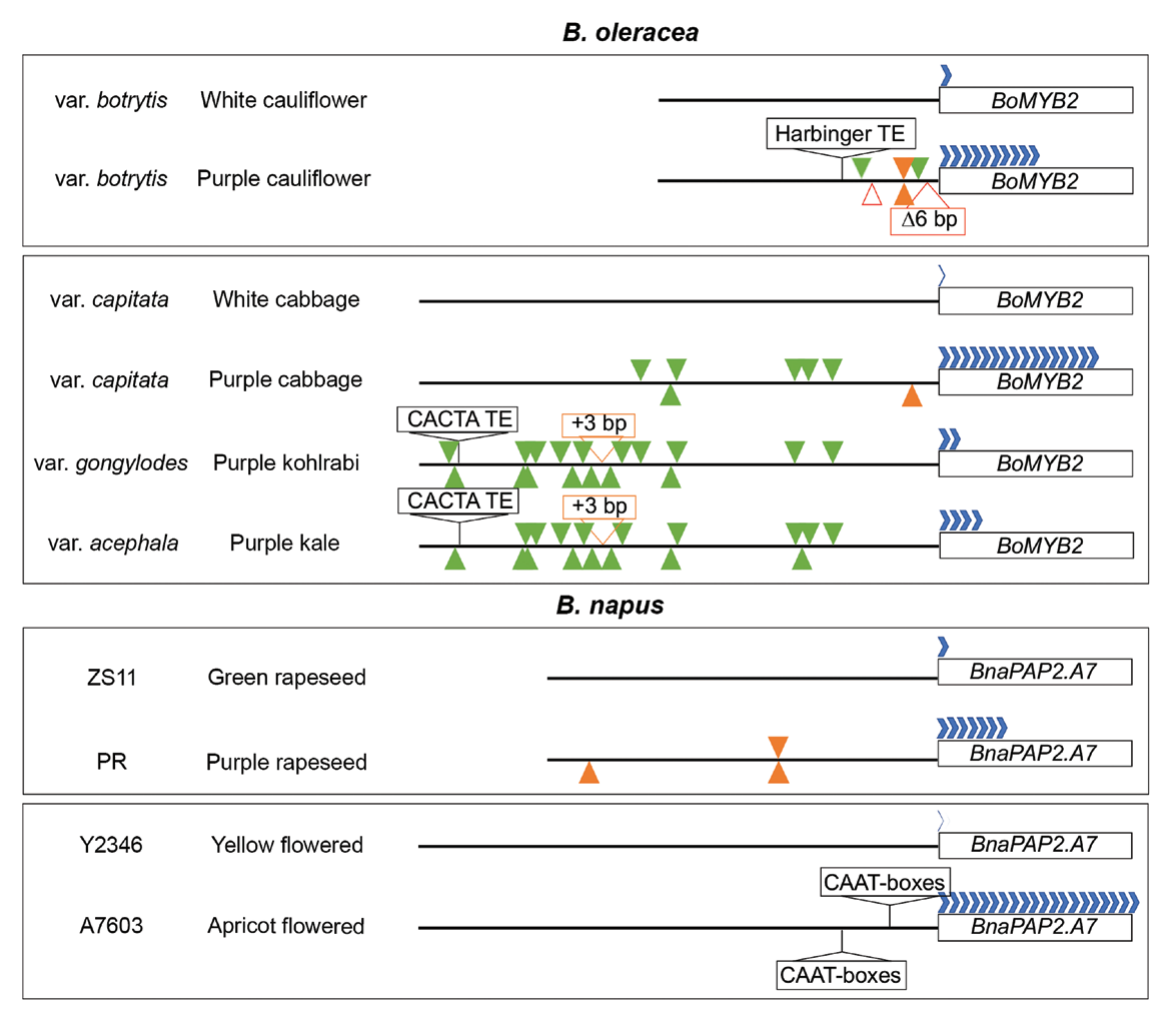

Some MYBs have a dual role in regulating floral traits. The *Petunia hybrida* R2R3-MYB transcription factor PH4 co-regulates volatile emission and vacuolar acidification, with the latter contributing to petal hue (Cna’ani *et al.*, 2015). In addition, *Arabidopsis thaliana* PAP1 (AtPAP1), known to activate phenylpropanoid pathway genes (Tohge *et al.*, 2005), promotes anthocyanin pigmentation and scent production when ectopically expressed in *Rosa hybrida* and *P. hybrida* (Zvi *et al.*, 2008, 2012). Interestingly, crosstalk has been noted between the carotenoid and anthocyanin pathways in *B. napus* (Liu *et al.*, 2020). The biosynthesis of both carotenoid and anthocyanin is regulated by REDUCED CAROTENOID PIGMENTATION 1 in *Erythranthe lewisii* (Sagawa *et al.*, 2016). The evolution of these floral traits is coupled due to a shared role in pollinator attraction and, in some cases, shared precursors. Ye *et al.* (2022) have made an important step in the journey towards a synthesized understanding of how showy floral traits are genetically encoded.
